# High-Quality Epitaxial Cobalt-Doped GaN Nanowires on Carbon Paper for Stable Lithium-Ion Storage

**DOI:** 10.3390/molecules29225428

**Published:** 2024-11-18

**Authors:** Peng Wu, Xiaoguang Wang, Danchen Wang, Yifan Wang, Qiuju Zheng, Tailin Wang, Changlong Sun, Dan Liu, Fuzhou Chen, Sake Wang

**Affiliations:** 1 College of Sino-German Science and Technology, Qingdao University of Science and Technology, Qingdao 266061, China; peng2004wu@163.com (P.W.); xgwang689@163.com (X.W.); wangdanchen1232024@163.com (D.W.); 13153710919@163.com (Y.W.); 2 School of Materials Science and Engineering, Qilu University of Technology, Jinan 250353, China; qlzhengqj@163.com (Q.Z.); tawangtailin@163.com (T.W.); 3 Key Laboratory of Chemical Engineering in South Xinjiang, College of Chemistry and Chemical Engineering, Tarim University, Alar 843300, China; happysunchanglong@126.com; 4 School of Materials Science and Engineering, Dongguan University of Technology, No. 1, Daxue Rd, Songshan Lake, Dongguan 523403, China; liudan@dgut.edu.cn; 5 College of Science, Jinling Institute of Technology, Nanjing 211169, China

**Keywords:** GaN, doped, CVD method, lithium-ion batteries, DFT

## Abstract

Due to its distinctive structure and unique physicochemical properties, gallium nitride (GaN) has been considered a prospective candidate for lithium storage materials. However, its inferior conductivity and unsatisfactory cycle performance hinder the further application of GaN as a next-generation anode material for lithium-ion batteries (LIBs). To address this, cobalt (Co)-doped GaN (Co-GaN) nanowires have been designed and synthesized by utilizing the chemical vapor deposition (CVD) strategy. The structural characterizations indicate that the doped Co elements in the GaN nanowires exist as Co^2+^ rather than metallic Co. The Co^2+^ prominently promotes electrical conductivity and ion transfer efficiency in GaN. The cycling capacity of Co-GaN reached up to 495.1 mA h g^−1^ after 100 cycles. After 500 cycles at 10 A g^−1^, excellent cycling capacity remained at 276.6 mA h g^−1^. The intimate contact between Co-GaN nanowires and carbon paper enhances the conductivity of the composite. Density functional theory (DFT) calculations further illustrated that Co substitution changed the electron configuration in the GaN, which led to enhancement of the electron transfer efficiency and a reduction in the ion diffusion barrier on the Co-GaN electrode. This doping design boosts the lithium-ion storage performance of GaN as an advanced material in lithium-ion battery anodes and in other electrochemical applications.

## 1. Introduction

Lithium-ion batteries (LIBs) are considered one of the most promising energy storage devices [1,2,3]. However, commercial anodes based on graphite materials fail to satisfy practical utilization requirements due to their limited capacity and rate performance [4]. Metal nitrides (MNs) have emerged as potential alternatives [5,6,7,8], but their slow charge transport and poor cycling stability hinder their widespread adoption as anode materials [9]. As a result, research on new anode materials with enhanced structure stability and ion transport kinetics is crucial for achieving high-rate performance and cycling stability in LIBs [10,11]. Gallium nitride (GaN) is a strong candidate material for LIBs owing to its excellent structure stability and lithium storage mechanism [12,13]. Nevertheless, the rate performance of GaN is often restricted by inadequate ion transfer kinetics. Moreover, the low anode capacity of unsubstituted GaN (189 mA h g^−1^) remains a significant challenge for broader applications [14].

Recent research on improving the conductivity and ion transfer in GaN-based materials has mainly focused on morphological and structural modifications. Common strategies, such as nanostructuring and surface graphitization, have reinforced the lithium storage performance and the kinetics of GaN-based composite anodes. However, the lithium storage properties of pure GaN are hindered by its intrinsically low charge transfer efficiency, and improvements to GaN at this point are still challenging [15]. One potential approach to alleviate this shortcoming is to regulate the mobility of electrons enhancing lithium-ion storage kinetics in GaN-based materials for advanced LIBs [16].

The electron configurations of atoms in GaN control the diffusion efficiency during electrochemical reactions and determine the rate performance of GaN anode materials [17]. Electron density engineering has been proposed as an effective strategy to improve ion diffusion efficiency [18]. Consequently, designing the electronic structure of GaN anodes is crucial. In particular, many studies have proven that metal cationic substitution is an efficient strategy for regulating electron mobility and charge transfer efficiency in anode materials [19]. For example, Fe-doped GeO_2_ introduces active sites for lithium storage and enhances conductivity, resulting in ultra-long cycling stability [20]. The Fe-doped ZnS materials demonstrated an impressive capacity retention of 651 mA h g^−1^ with 94% of the capacity reserved [21]. Among the various cation substitution candidates, cobalt (Co) stands out because it is easy to access, inexpensive, and demonstrates superior electrical properties. Co cation substitution has been shown to enrich active sites for lithium insertion and optimize the adsorption energy of lithium ions [22,23]. Moreover, Co cation substitution activates the lithium storage processes by creating an increased number of active sites [24,25]. Considering the electron configuration of the Co element, Co cation substitution is an efficient way to enhance ion diffusion in GaN-based anodes. However, studies on the reorganization of the electronic structure of GaN through Co cation substitution for optimized electrochemical performance remain limited.

In this work, Co-substituted GaN (Co-GaN) nanowires on carbon paper were designed and synthesized via a facile chemical vapor deposition (CVD) strategy. Electrochemical measurements and DFT calculations confirmed improvement of the lithium-ion storage performance in the Co-substituted GaN. The orbital hybridization between the Co and N elements revealed a significant decrease in the bandgap and increased electron delocalization. Consequently, Co substitution enhanced electron conductivity and ion transfer in GaN, thus achieving high-rate performance with stable cycling capacity. The lithium storage capacity of the Co-GaN electrode reached 813.2 mA h g^−1^ after 200 cycles at a current density of 0.1 A g^−1^. This electron density reorganization engineering through Co cation substitution offers profound insights into designing high-performance lithium storage anode materials and in other realms.

## 2. Results and Discussion

### 2.1. Morphology Characterization

**Figure 1:**
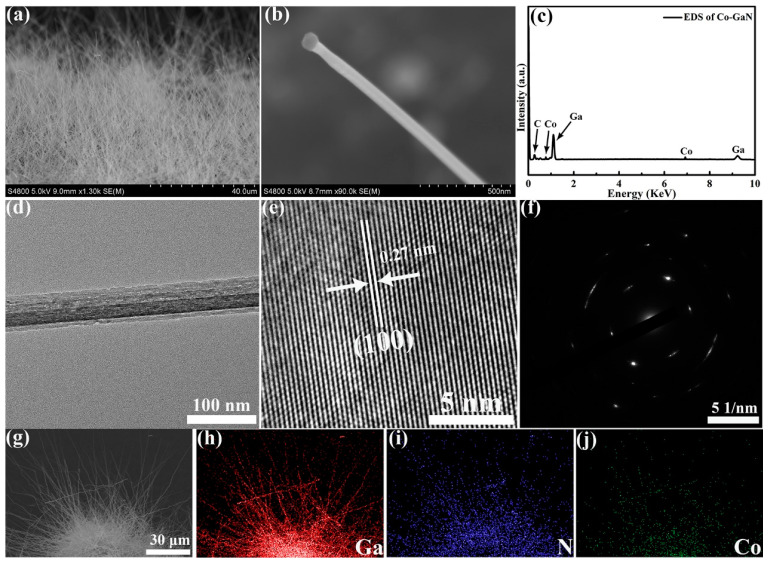
(**a**,**b**) SEM images at different magnifications. (**c**) Energy-dispersive X-ray (EDX) elemental test. (**d**) TEM image. (**e**) High-resolution TEM images. (**f**) SAED images. (**g**–**j**) EDS mapping analysis of Co-GaN.

Co-GaN nanowires were synthesized using the CVD method. The scanning electron microscopy (SEM) image in Figure 1a illustrates the nanowire morphology of Co-GaN. The high-resolution SEM image (Figure 1b) reveals that the surface of Co-GaN remained smooth and showed no significant changes after cobalt substitution. The energy-dispersive X-ray (EDX) analysis (Figure 1c) confirmed the presence of Co, Ga, and N elements in the nanowires, with atomic percentages of approximately 5.3%, 40.5%, and 54.2%, respectively. Elemental mapping (Figure 1g–j) directly showed the uniform distribution of Co, Ga, and N, indicating successful Co doping in the nanowires. The transmission electron microscope (TEM) image (Figure 1d) shows the fine structure of Co-GaN nanowires with a diameter of around 50 nm. The high-resolution TEM (HRTEM) image in Figure 1e depicts the fine crystallization structure of Co-GaN with an interplanar spacing of 2.76 Å. Selected area electron diffraction (SAED) (Figure 1f) confirmed that the Co-GaN nanowires grew along the (100) direction. The fine crystal structure of Co-GaN, which is crucial for achieving high-rate performance in lithium anodes, was further confirmed by means of HRTEM imaging [26]. Furthermore, interface integration between the Co-GaN nanowires and the carbon paper, which can greatly reduce the lithium-ion transport distance and ensure efficient electron conductivity, was confirmed. These designed structural characteristics and synergistic effects contribute to the enhanced electrochemical performance.

### 2.2. Structure Characterization

**Figure 2:**
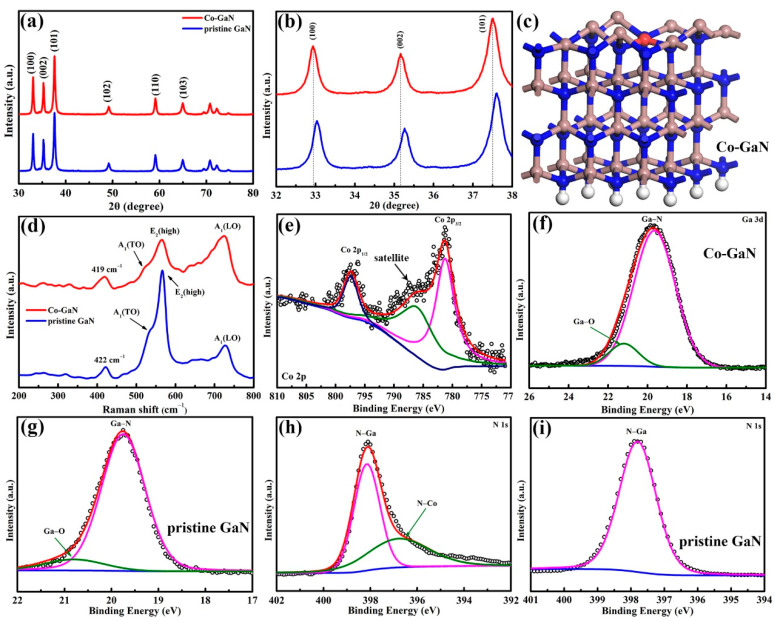
(**a**) XRD patterns, (**b**) amplified XRD patterns from 32° to 38°, and (**c**) the corresponding schematic structure model of Co-GaN (White ball for hydrogen atom, pink ball for gallium atom, blue ball for nitrogen atom and red ball for cobalt atom). (**d**) Raman spectra, (**e**) Co 2p, (**f**,**g**) Ga 3d, and (**h**,**i**) N 1s XPS spectra of GaN and Co-GaN, respectively.

The crystallographic structure of both pristine GaN and Co-GaN was further characterized using X-ray diffraction (XRD). As depicted in Figure 2a, all peaks for both pristine GaN and Co-GaN nanowires were indexed to a hexagonal crystal system [C 6v4 P63mc, JCPDS: No. 50-0792]. The peak intensity result shows that Co doping has limited effect on Co-GaN crystallinity. Figure 2b shows that the primary peaks of (100), (002), and (101) in Co-GaN shifted to higher angles compared to the GaN pattern. This shift is attributed to the substitution of smaller Ga ions with Co ions according to the Bragg equation (2d sin θ = kλ). Additionally, the full width at half-maximum (FWHM) of the Co-GaN nanowire peaks increased compared to that for the GaN pattern, illustrating the presence of the Co dopant, which changed the crystal structure of GaN (Figure 2c). No impurity peaks were found in the Co-GaN patterns, allowing us to infer that there was no pollution in the Co-doped sample. In the Raman spectra (Figure 2d), two peaks were found at 534 cm^−1^ and 569 cm^−1^. The 534 cm^−1^ peak corresponds to the A_1_ mode of GaN, while the 569 cm^−1^ peak indicates the E_2_ mode of GaN [27]. In the Co-GaN sample, a broad peak located at 664.7 cm^−1^ was observed, which was caused by the defect states introduced by Co doping [28]. The similarity in Raman spectra between the GaN and Co-doped GaN nanowires confirms that the origin microstructure of GaN was well-preserved after Co doping. This well-maintained structure facilitates efficient electron transport, which is essential for elevating the rate performance of the active Co-GaN nanowires.

X-ray photoelectron spectroscopy (XPS) was implemented to examine the chemical bonding of Co, Ga, and N in the Co-GaN nanowires and to reveal the change in the chemical environment after the Co doping. The XPS spectra of the Co 2p are shown in Figure 2e. These spectra are deconvoluted into two primary peaks. The peaks at 781.3 eV and 797.5 eV correspond to the Co 2p_3/2_ and Co 2p_1/2_ levels, respectively. The Co 2p_3/2_ spectrum possesses a satellite peak at 785.4 eV [29], and the observed energy separation (ΔE) reveals the exchange interaction energy. The spin-orbit splitting of 16.2 eV for the Co 2p doublet indicates a blended valence state of Co of approximately 2+ and 3+. The existence of Co^2+^ and Co^3+^ indicates that Co donated electrons to GaN and changed the electron distribution in GaN. Based on the Co 2p spectra, the Co concentration in the Co-GaN nanowires was calculated to be 4.6% (atomic percent). These results confirm that the Co element was successfully doped into the GaN nanowires. Figure 2f shows the high-resolution core-level spectra of Ga 3d for both GaN and Co-GaN nanowires. The Ga XPS spectra were deconvoluted into a peak located at 19.5 eV, indicating the Ga-N bond, and a peak located at 21.2 eV, indicating the Ga-O bond. The presence of Ga-O bonds is due to the slight oxidation during the nitridation process of Co-GaN nanowire formation. Notably, Co doping reduced the percentage of Ga-O bonds, as reflected by the diminished Ga-O peak intensity in Figure 2g. This reduction is likely due to Co’s lower electronegativity increasing the electron density around Ga atoms. The negative shift in the Ga 3d binding energy confirms enhanced coupling between Co and GaN due to Co doping. The N 1s spectra of pristine GaN (Figure 2h) and Co-GaN nanowires (Figure 2i) reveal N-Ga bonds at 397.9 eV [30]. Additionally, in the Co-GaN nanowires, the characteristic binding energy for N-Co bonds at 396.4 eV was observed, further confirming the incorporation of Co into the nanowires. The Co 2p and N 1s spectra together provide strong evidence for successful Co doping in the Co-GaN nanowires.

### 2.3. Electrochemical Analysis

**Figure 3:**
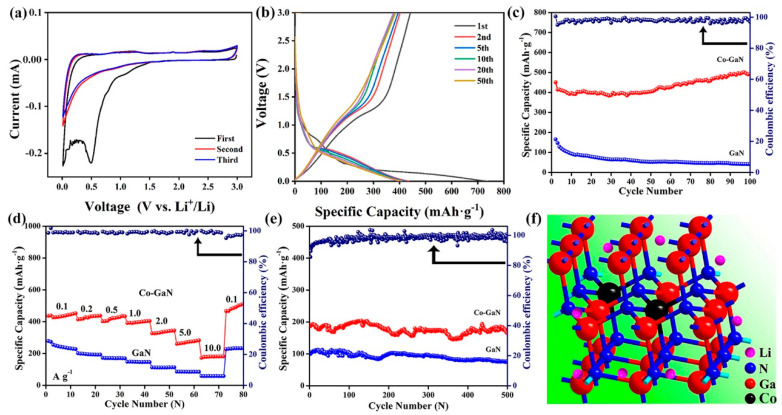
Electrochemical test results of the Co-GaN nanowires: (**a**) CV tests at a scan rate of 0.1 mV s^−1^. (**b**) Galvanostatic charge and discharge (GCD) tests. (**c**) Cycling performance test. (**d**) Rate performance test of GaN and Co-GaN nanowire electrodes. (**e**) The long cycling at a high current density of 10.0 A g^−1^. (**f**) Schematic illustration of lithium transfer channel.

Figure 3a shows the cyclic voltammetry (CV) test result for the Co-doped GaN. In the first cycle, the broad peaks observed at 0.5 and 1.1 V denote the formation of the solid electrolyte interphase (SEI) layer [31]. In the subsequent cycles, a peak located at 0.85 V corresponds to the interaction between Li and N [32]. The following CV curves overlaid with the former one indicate excellent stability in the cycling processes, with the similarity in peak positions suggesting that Co doping does not change the lithium storage mechanism. The cycling capacity and stability of Co-GaN are shown in Figure 3b. The irreversible capacity was 747.2 mA h g^−1^ in the first discharge cycle. The irreversible capacity reduction in the first discharge and charge process may have been induced by the consumption of lithium ions forming SEI layers [33]. In subsequent cycles, the overlapping galvanostatic charge and discharge (GCD) profiles suggest stable structure retention and the steady lithium storage mechanism of the Co-GaN sample [34]. The cycling capacity remained at 495.1 mA h g^−1^, with the Coulombic efficiency maintained at ~100% after 100 cycles (Figure 3c). The reasons for this elevated lithium-ion storage performance are the improved ion diffusion kinetics and the lowered ion diffusion barrier after Co doping. As shown in Figure 3d, the Co-GaN electrode achieved rate capacities of 452.9, 431.1, 426.5, 403.4, 337.3, and 276.6 mA h g^−1^ at current densities of 0.1, 0.2, 0.5, 1.0, 2.0, and 5.0 A g^−1^, respectively. Even at the high current density of 10.0 A g^−1^, the electrode demonstrated a specific capacity of 181.5 mA h g^−1^, significantly better than the capacity of the GaN sample. After the current density changed back to 0.1 A g^−1^, the specific capacity returned to 455.8 mA h g^−1^, reflecting excellent reversibility in lithium storage. The excellent rate performance presented by the Co-GaN sample is due to the improved ion diffusion and enhanced electron conductivity [35]. The ultralong cycling performance of Co-GaN was also tested at the high rate of 10.0 A g^−1^ (Figure 3e). After 500 cycles, the cycling capacity remained at 167.7 mA h g^−1^, indicating that Co doping significantly improved the lithium-ion activity in the Co-GaN nanowires. Figure 3f provides a direct model of the lithium diffusion channel in the Co-doped GaN sample; this model illustrates the decreased lithium diffusion length and the utility of the Co dopant [36]. The increased cycling and rate performance demonstrate the excellent electrochemical properties of Co-GaN.

**Figure 4:**
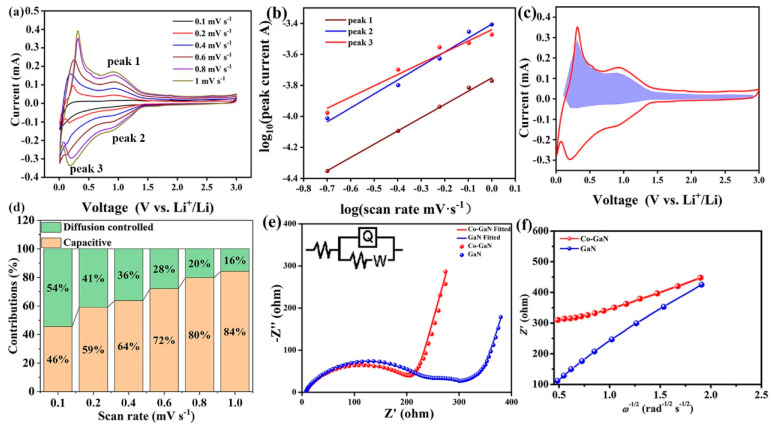
(**a**) CV tests of Co-GaN at various scan rates. (**b**) Determination of the calculated *b* value. (**c**) Pseudocapacitive contribution at 1.0 mV s^−1^. (**d**) Pseudocapacitive contribution illustration at the scan rate of 1.0 mV s^−1^. (**e**) Electrochemical impedance spectra (EIS) with the fitted Nyquist plots and the equivalent circuit of the GaN and Co-GaN electrodes. (**f**) The calculation of relationships between Z’ and ω^−1/2^.

The CV curves for Co-GaN under various scan rates are depicted in Figure 4a. The CV curves at various scan rates have similar shapes, indicating the stable lithium storage mechanism and small polarization during the lithium storage reaction under high current densities [37]. The lithium storage behavior and the pseudocapacitive contribution of Co-GaN can be calculated according to Equation (1),

*i* = *av^b^* (1)

where *i* is the current, and *v* is the scan rate. Constants *a* and *b* denote the lithium storage behavior, which can be calculated from the data under different scan rates [38]. The calculated *b* value (Figure 4b) shows the lithium storage behavior containing both diffusion contributions and capacitive contributions. These two contributions can be calculated from the capacitive effect (*k*_1_*v*) [39]. In the Co-GaN nanowire electrode (Figure 4c), over 40% of the total capacity results from the capacitive process (the purple region).

The two different capacitive contributions can be calculated from the various CV plots under various scan rates. The calculated contributions of Co-GaN at different scan rates are shown in Figure 4d. The pseudocapacitive contribution in Co-GaN increases with an increasing scan rate, which is consistent with previous test results [40]. To further assess the elevating effect of Co cation substitution on ion diffusion, Nyquist plots were analyzed (Figure 4e). The semicircles at high-to-medium frequencies denote the charge-transfer resistance (Rct), while the inclined lines at low frequencies correspond to the mass-transfer resistance [41]. After the data were fitted to an equivalent circuit (inset of Figure 4e), the Rct of the Co-GaN electrode (204.6 Ω) was significantly lower than that of GaN (297.5 Ω), indicating improved conductivity and enhanced ion diffusion efficiency after Co doping [42]. In the low-frequency region, the slope of Co-GaN was steeper than that of the pristine GaN sample, confirming improved lithium-ion mobility and a more favorable pore structure or diffusion pathway in the Co-GaN electrode [43]. According to Equation (2),

*D*_Li+_ = *R*^2^*T*^2^/2*A*^2^*n*^4^*F*^4^*C*^2^*σ*^2^, (2)

where *T*, *F*, and *R* are constants that stand for the absolute temperature, Faraday’s constant, and the gas constant. *A* is the area of the electrode, and C is the lithium-ion molar concentration. The Warburg factor (*σ*) can be calculated using Equation (3):

*Z_real_* = *R_e_* + *R_c_*_t_ + *σω*^−1/2^ (3)

In Figure 4f, the calculated *σ* values for Co-GaN and GaN are 121.6 and 87.3; the *σ* value for Co-GaN is much higher than that for GaN (87.3). The lithium-ion diffusion coefficient (*D*_Li+_) for Co-GaN is 4.9 × 10^−12^ cm^2^ s^−1^ according to the calculation, which is much higher than that for GaN (6.2 × 10^−13^ cm^2^ s^−1^). The enhanced diffusion coefficient indicates elevated ion diffusion efficiency and enhanced charge transfer kinetics in Co-GaN brought about by the Co doping. Moreover, one can infer from the electrochemical results that there was no conversion or alloy lithium storage mechanism in the Co-doped GaN, further illustrating the derivation of the stable lithium-ion storage performance. These results also indicate that Co doping had a minimal impact on the fundamental lithium-ion storage mechanism, highlighting the enhanced conductivity and improved ion diffusion kinetics achieved through Co doping.

### 2.4. First-Principles Analysis

**Figure 5:**
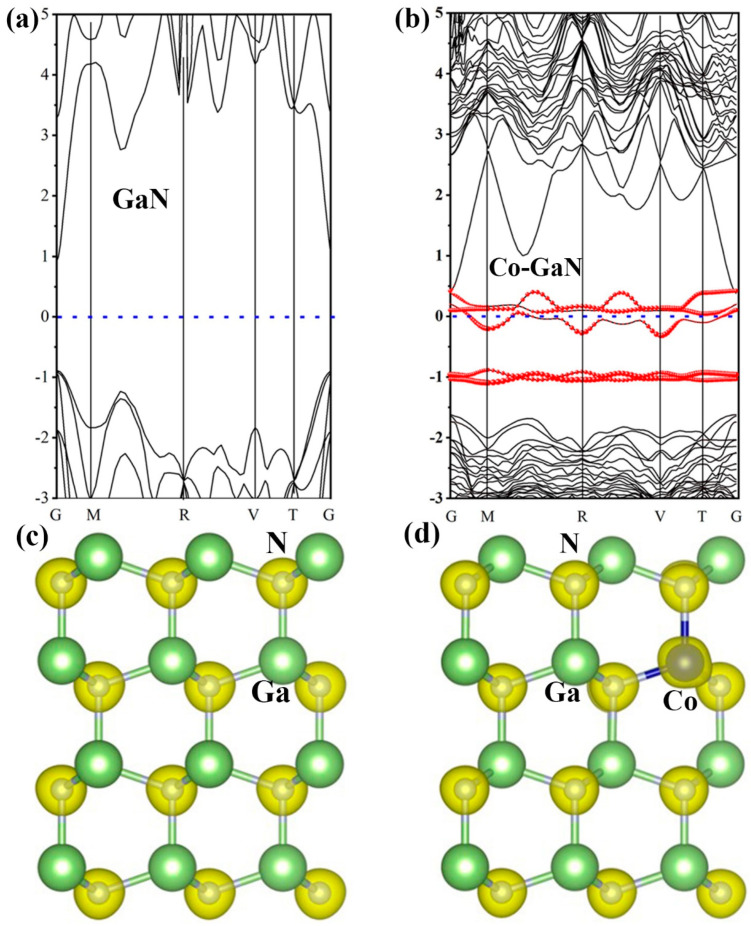
Band structure of (**a**) GaN and (**b**) Co-GaN. (**c**,**d**) Differences in charge density of GaN and Co-GaN.

To determine the chemical origin of the enhanced lithium storage performance in Co-GaN, DFT analysis was utilized to study the effects of Co doping on the electronic configuration of Co-GaN. The calculated band structures based on the models of GaN and Co-GaN are illustrated in Figure 5a,b. In Figure 5a, discrete energy levels can be observed in the pristine GaN band structure, resulting in semiconductor behavior in GaN [13]. For Co-GaN, the band structure indicates a continuous energy band near the Fermi level (Figure 5b) compared with the GaN band structure. Therefore, Co doping results in stronger conductivity brought about by the additional orbits around the Fermi level. These DFT results indicate that Co doping effectively modulated the electron density in the GaN. Therefore, the change in the band structure accelerated the electrochemical kinetics for lithium-ion storage. Furthermore, a charge density analysis was conducted to provide a better understanding of the charge transfer produced by Co doping (Figure 5c,d). The results showed that charge density accumulates at the Co atom side after Co doping. After the Co doping, the bond length between Co and N was shortened due to the strong covalent link. Due to the stronger electronegativity of Co, the electrons accumulate near the Co atom while the Ga atoms possess the electron depletion region. These results definitively demonstrate the uneven distribution of charge density in Co-GaN (Figure 5d). Therefore, based on the DFT calculation, the enhanced electrochemical properties and improved lithium storage performance are derived from the significant charge transfer induced by Co doping. This pronounced charge transfer facilitates more efficient lithium-ion storage, contributing to the superior electrochemical performance of Co-GaN.

## 3. Materials and Methods

### 3.1. Synthesis of Co-GaN Loaded on Carbon Paper

Co-GaN nanowires on carbon paper were synthesized using a designed chemical vapor deposition (CVD) method. In this process, 0.5 g of Ga_2_O_3_ and 0.32 g of CoCl_2_ were placed in an alumina boat and then set at the center of a tube furnace. A carbon paper substrate was positioned approximately 2 inches from the alumina boat. The tube was initially purged with nitrogen (N_2_) to remove any residual air before initiating the growth process. After evacuation, the tube was heated to 1100 °C at a rate of approximately 8 °C/min using a local heater while maintaining a constant N_2_ flow acting as a protective gas. Once the furnace reached the set temperature, ammonia (NH_3_) was introduced at a flow rate of 100 sccm to trigger the reaction. After the growth was completed, the furnace was cooled under continuous N_2_ flow.

### 3.2. Electrochemical Measurements

The lithium storage performance was evaluated using CR2016-type coin cells. The assembly was conducted in an argon-filled glove box. Carbon paper coated with Co-GaN nanowires (12 mm in diameter) was used as the anode, with an average loading density of approximately 1.9 mg cm^−2^. A lithium metal cathode, along with pristine GaN and Co-GaN electrodes, was electronically separated by glass microfiber filters (Whatman GF/D), which were saturated with an electrolyte solution of 1 M LiPF_6_ in a 1:1:1 volumetric mixture of ethylene carbonate, dimethyl carbonate, and diethyl carbonate.

The electrochemical performance was measured using an EWARE battery testing system and a CHI660D electrochemical workstation (Shanghai CH Instruments Co., Shanghai, China). Electrochemical impedance spectroscopy (EIS) was performed with a frequency range of 0.01 Hz to 1 MHz and at a 5 mV amplitude signal.

### 3.3. Measurement and Characterization

The structure and morphology of pristine GaN and Co-GaN nanowires were characterized using scanning electron microscopy (SEM, SigmaHD, Cambridge, UK). Powder X-ray diffraction (XRD) tests were conducted on a Rigaku D/MAXRB diffractometer, over a 2θ range from 20° to 80°, utilizing Cu Kα radiation (λ = 0.15 nm) (BRUKER D8 ADVANCE, Karlsruhe, Germany). Transmission electron microscopy (TEM) and high-resolution TEM (HRTEM) images were obtained with a JEOL JEM-F200 microscope operated at an acceleration voltage of 200 kV. Raman spectra were recorded using 532 nm laser excitation on a DXR2 micro-Raman spectrometer (Thermo Fisher, Carlsbad, CA, USA) equipped with an Olympus BX 41 optical microscope. X-ray photoelectron spectroscopy (XPS, Kratos Analytical Ltd., Manchester, UK) was conducted to verify the surface’s chemical composition and electron configuration.

### 3.4. Density Functional Theory Calculation

We performed first-principles calculations based on density functional theory (DFT) using the Vienna Ab-initio Simulation Package (VASP). The generalized gradient approximation (GGA) with the Perdew–Burke–Ernzerhof (PBE) functional was employed for the exchange-correlation potential. A plane-wave energy cutoff of 500 eV was applied. Structural relaxations were carried out until the forces were below 0.01 eV/Å, and the energy convergence criterion was set to 10^−6^ eV per atom. To simulate Co doping, a Ga atom was substituted with a Co atom, resulting in a Ga23Co1N24 bulk structure. For comparison, a GaN bulk supercell containing 48 atoms was also constructed, with an expansion coefficient of 2 × 3 × 2. The Brillouin zone was sampled using a 3 × 2 × 3 Monkhorst–Pack k-point mesh. The valence electrons included the 2s and 2p orbitals of N, the 4s and 4p orbitals of Ga, and the 4s and 3d orbitals of Co.

## 4. Conclusions

In summary, Co-GaN nanowires were designed and synthesized via the facile CVD method. An XPS analysis confirmed that Co doping effectively modulated the electronic properties of the GaN nanowires. Compared to pure GaN, the optimized Co-GaN nanowires exhibited significantly higher cycling capacity, enhanced rate performance, and superior cycling stability. More importantly, a DFT analysis revealed the emergence of hybridized electronic states after Co doping, which improved both the conductivity and lithium-ion diffusion. An electrochemical analysis further validated the exceptional lithium storage performance of the Co-GaN nanowires. This design strategy provides a practical approach for activating GaN by regulating its electronic structure and provides a profound understanding about the influence of transition metal doping at the atomic level.

## Data Availability

The data that support the findings of this study are available from the corresponding author upon reasonable request.
